# Genomic Comparison of *Lactobacillus casei* AP and *Lactobacillus plantarum* DR131 with Emphasis on the Butyric Acid Biosynthetic Pathways

**DOI:** 10.3390/microorganisms9020425

**Published:** 2021-02-19

**Authors:** Widodo Widodo, Aditya Lutfe Ariani, Donny Widianto, Dietmar Haltrich

**Affiliations:** 1 Graduate School of Biotechnology, Universitas Gadjah Mada, Yogyakarta 55281, Indonesia; adityalufte2103@gmail.com (A.L.A.); donny@ugm.ac.id (D.W.); 2 Faculty of Animal Science, Universitas Gadjah Mada, Yogyakarta 55281, Indonesia; 3 Department of Microbiology, Faculty of Agriculture, Universitas Gadjah Mada, Yogyakarta 55281, Indonesia; 4 Food Biotechnology Laboratory, University of Natural Resources and Life Sciences, 1190 Vienna, Austria; dietmar.haltrich@boku.ac.at

**Keywords:** butyric acid, *Lactobacillus*, butyrate kinase, phosphate butyryl transferase

## Abstract

Butyric acid is known to possess anticarcinogenic and antioxidative properties. The local lactic acid bacteria (LAB) strains *Lactobacillus casei* AP isolated from the digestive tract of healthy Indonesian infants and *L. plantarum* DR131 from indigenous fermented buffalo milk (*dadih*) can produce butyric acid in vitro. However, the genes and metabolic pathways involved in this process remain unknown. We sequenced and assembled the 2.95-Mb *L. casei* AP and 4.44-Mb *L. plantarum* DR131 draft genome sequences. We observed that 98% of the 2870 protein-coding genes of *L. casei* AP and 97% of the 3069 protein-coding genes of *L. plantarum* DR131 were similar to those of an *L. casei* strain isolated from infant stools and an *L. plantarum* strain in sheep milk, respectively. Comparison of the genome sequences of *L. casei* AP and *L. plantarum* DR131 led to the identification of genes encoding butyrate kinase (*buk*) and phosphotransbutyrylase (*ptb*), enzymes involved in butyric acid synthesis in *L. casei* AP. In contrast, a medium-chain thio-esterase and type 2 fatty acid synthase facilitated butyric acid synthesis in *L. plantarum* DR131. Our results provide new insights into the physiological behavior of the two LAB strains to facilitate their use as probiotics.

## 1. Introduction

Lactic acid bacteria (LAB) have been widely used for the fermentation and production of a variety of food items for human and animal consumption. These bacteria enhance the flavor, texture, nutritional value, and safety of fermented food items. In addition, specific strains of LAB are known to have probiotic potential owing to their beneficial effects on the health of consumers [1]. Previous studies have reported that the beneficial health effects of LAB are associated with its ability to produce many bioactive compounds, including exopolysaccharides [2,3], riboflavin [4], gamma-aminobutyric acid (GABA) [5], and short-chain fatty acids (SCFAs) [6]. Recent advances in microbial genomics, such as genome sequencing and functional genomic analyses, have broadened our knowledge regarding the diversity and evolution of LAB strains. Further, they have aided in the analysis of important food traits, such as flavor formation, sugar metabolism, stress response, adaptation, and molecular-level interactions [7].

Butyric acid (butyrate) is an SCFA synthesized by the microflora present in the intestine [8]. Butyrogenesis (or butyrate production) has been widely researched in several gut-related studies [9,10] as well as in biotechnology [11]. In bacteria, butyric acid can be synthesized either directly from carbohydrates via butyrate kinase or indirectly from acetate, succinate, and lactate via butyryl-coenzyme A (CoA): acetate-CoA transferase, succinyl-CoA synthetase, and lactate dehydrogenase, respectively, together with butyrate kinase [12]. Due to its wide range of beneficial roles, including as an essential energy source for epithelial cells of the large intestine and for the prevention of inflammatory bowel disease and colorectal cancer [13], butyric acid has huge potential for use in probiotics. However, studies related to butyric acid biosynthetic pathways in *L. casei* species have been limited to their physiology. Therefore, we selected two local LAB strains, namely *Lactobacillus casei* AP, isolated from the digestive tract of a healthy infant aged <1 month, and *L. plantarum* DR131, obtained from indigenous fermented buffalo milk (*dadih*) in Indonesia. Both *L. casei* AP and *L. plantarum* DR 131 can potentially be developed into fermented dairy products as a health food. Widodo et al. [14] reported that the use of human-origin *L. casei* AP in fermented dairy products could reduce hyperglycemia and cholesterol in Sprague Dawley rats. Further, *L. plantarum* DR131 has also been used to produce fermented buffalo milk in Indonesia.

Kusmiyati et al. [15] demonstrated that *L. casei* AP produced butyric acid in media containing inulin, whereas Pessione et al. [6] reported that *L. plantarum* was capable of producing butyric acid. Based on these findings, we explored the genes, pathways, and mechanisms involved in butyric acid synthesis using genome sequencing and annotation to verify the experimental outcomes in both strains.

## 2. Materials and Methods

### 2.1. Bacterial Strain Identification

*L. casei* AP was isolated from the fecal sample of a healthy infant [16] and *L. plantarum* DR131 was isolated from indigenous fermented buffalo milk (*dadih*). These strains were identified by their 16S rRNA using 27 F and 1492 R primers. A DNA fragment of approximately 500–1000 kb was amplified using the Bio-Rad Thermal Cycler (Bio-Rad, Hercules, CA, USA). Polymerase chain reaction (PCR) was performed under the following conditions: initial denaturation at 96 °C for 4 min followed by 30 cycles of denaturation at 94 °C for 1 min, annealing at 52 °C for 1 min 50 s, and a final extension at 68 °C for 8 min. The PCR products were analyzed using 1.0% (*w/v*) agarose gel electrophoresis (Bio-Rad) in 1× Tris/borate/ethylenediaminetetraacetic acid buffer at 100 V for 30 min and visualized on a gel documentation system (BioDocAnalyze; Biometra GmbH, Gottingen, Germany). The purified PCR products were sequenced using 16S rRNA primers. Whole-genome sequences were used for similarity searches against the NCBI GenBank database using the Basic Local Alignment Search Tool (BLAST) program available at the website (http://blast.ncbi.nlm.nih.gov/BLAST.cgi (accessed on 6 January 2021)).

### 2.2. Genome Sequencing and Assembly

The DNA from *L. casei* AP and *L. plantarum* DR131 was extracted using the Presto^TM^ Mini gDNA Bacteria Kit (Geneaid) according to the manufacturer’s instructions. The final DNA concentration was determined using the Qubit 2.0 Fluorometer (Life Technology, Carlsbad, CA, USA). Whole-genome sequencing was performed on the Illumina platform using the massively parallel sequencing technology. Paired-end (PE) adapters ligated to A-tailed fragments and PCR amplified with a 500-bp insert and a mate-pair (MP) library with a 5-kb insert were used for the construction of the genome library at Novogene Bioinformatics Technology Co., Ltd. (Beijing, China). The PE and MP libraries were sequenced on the Illumina HiSeq platform using the PE150 strategy. We used our own compiling pipeline to filter the Illumina PCR adapter reads and low-quality reads from the PE and MP libraries. All good quality PEs were assembled into several scaffolds using the SOAPdenovo genome assembler (http://soap.genomics.org.cn/soapdenovo.html (accessed on 6 January 2021)) [17,18]. The reads were filtered during the gap-closing step.

#### 2.2.1. Genome Component Prediction

Genome component prediction included the prediction of coding genes, repetitive sequences, and non-coding RNA. For bacteria, we used the GeneMarkS (http://topaz.gatech.edu/GeneMark/ (accessed on 6 January 2021)) program to retrieve the related coding genes [19]. The interspersed repetitive sequences were predicted using RepeatMasker (http://www.repeatmasker.org/ (accessed on 6 January 2021)) [20]. Tandem repeats were analyzed by the tandem repeats finder (http://tandem.bu.edu/trf/trf.html (accessed on 6 January 2021)) [21]. Transfer RNA (tRNA) genes were predicted using tRNAscan-SE [22]. Ribosomal RNA (rRNA) genes were analyzed using rRNAmmer [18]. Small nuclear RNAs (snRNA) were predicted by BLAST against the Rfam database [23,24].

#### 2.2.2. Genome Function Annotation

Protein-coding genes were predicted using GeneMarkS (http://topaz.gatech.edu/GeneMark/ (accessed on 6 January 2021)) [25]. We used the following seven databases to predict gene functions: Gene Ontology [26], the Kyoto Encyclopedia of Genes and Genomes (KEGG) [27,28], Clusters of Orthologous Groups [29], the Non-Redundant Protein Database (NR) [18], Swiss-Prot [30], TrEMBL Uniport [31], Brenda enzymes (www.brenda-enzymes.org (accessed on 6 January 2021)), and MetaCyc (https://metacyc.org/ (accessed on 6 January 2021)). A whole-genome BLAST search (*E*-value < 1 × 10^−5^ [32], minimal alignment length > 40%) was performed against these seven databases.

## 3. Results

### 3.1. Genomic Characteristics of L. casei AP and L. plantarum DR131

The genomes of *L. casei* AP and *L. plantarum* DR131 were sequenced using the whole-genome shotgun strategy to produce clean data after filtering low-quality reads and reads with adapter contamination. First, the genome of *L. casei* AP was assembled using the SOAPdenovo (version 2.04) assembler to generate 79 contigs (>500 bp) with N50 of 83,564 base pairs (bp) [18,33] followed by assembly into 71 scaffolds (>500 bp) with N50 of 83,564 bp. The lengths of the scaffolds ranged from 557 bp to 277,281 bp. For *L. plantarum* DR131, 87 contigs (>500 bp) with N50 of 120,340 bp assembled into 67 scaffolds (>500 bp) with N50 of 128,698 bp were generated. The lengths of the scaffolds ranged from 503 bp to 262,440 bp. We could not assemble the scaffolds into chromosomes via K-mer analysis for *L. casei* AP and *L. plantarum* DR131. However, for *L. casei* AP, we obtained a draft genome sequence of 2.95 Mb as compared to the expected genome size of 3.05 Mb; this indicates that the scaffolds covered 96.72% of the whole genome with a K-mer depth of 85.87. In the case of *L. plantarum* DR131, we obtained a draft genome sequence of 4.44 Mb compared to the expected genome size of 4.47 Mb, indicating that the scaffolds covered 99.33% of the whole genome with a K-mer depth of 60.38. The G + C content of *L. casei* AP and *L. plantarum* DR131 was 46.34% and 44.42%, respectively.

### 3.2. Identification of the Butyric Acid Biosynthetic Pathways

We analyzed the genes involved in the butanoate metabolism (map00650) pathway in the genomes of *L. casei* AP and *L. plantarum* DR131. The genes encoding for butyrate kinase (*buk*) (locus = Scaffold14:33935:35056:+) and phosphotransbutyrylase (*ptb*) (locus = Scaffold14:33042:33938:+), enzymes responsible for butyric acid synthesis, were found in *L. casei* AP, but not in *L. plantarum* DR131. The results of KEGG annotation of the genes presumably involved in butanoate metabolism in *L. casei* AP are presented in Table 1.

After excluding these terminal genes and their respective butanoate metabolism pathways, we searched all the genes responsible for the enzymatic reactions involved in butyric acid production (www.brenda-enzymes.org (accessed on 6 January 2021); http://www.genome.jp/kegg/annotation/enzyme.html (accessed on 6 January 2021); https://metacyc.org/ (accessed on 6 January 2021)). Recently, Botta et al. [34] reported that butyrogenesis in *L. plantarum* occurs via the complementary activities of a medium-chain thio-esterase and type 2 fatty acid synthase (FASII). This pathway is also involved in the biosynthesis of hexanoic acid, octanoic acid, decanoic acid, dodecanoic acid, and tetradecanoic acid. The butyrogenic capability of *L. plantarum* varies based on the strain and is highly dependent on the substrate type, with glutamine/glutamate playing an important role. However, this has not been assigned to any specific metabolic pathway. A previous study by Botta et al. [34] linked the loss of the butyrogenic capability of *L. plantarum* to deleterious functional mutations in the enzyme glutamate decarboxylase and a glutamine ABC transporter. We found similar results for *L. plantarum* DR131, confirming that butyrogenesis can only occur via the complementary activities of a medium-chain thioesterase and FASII in *L. plantarum*. In addition, the evaluation of butanoate metabolism in *L. plantarum* DR131 revealed the involvement of two glutamate decarboxylase (*gad*) genes. GAD is the key enzyme involved in GABA biosynthesis [35] and is linked to stress resistance and pH homeostasis in the cytosol via protein translocation [36].

The results of the KEGG annotation of the genes involved in the proposed butanoate metabolism in *L. plantarum* DR131 are shown in Table 2.

By integrating the KEGG annotation of butanoate metabolism in *L. plantarum* DR131 and previously published GABA biosynthesis in *L. plantarum*, we proposed the potential involvement of the GABA pathway in *L. plantarum* DR131 in butanoate synthesis via an unknown mechanism. The results of KEGG annotation of the genes involved in butyric acid production can be attributed to the complementary activities of a medium-chain thio-esterase and FASII in *L. plantarum* DR131 (Table 3).

## 4. Discussion

LABs are widely used in the fermentation and production of a wide range of food products due to their ability to impart flavor and texture to the fermented products. In addition, specific LAB strains are known to have the ability to act as probiotics owing to their health-promoting effects in animals and humans [1]. The sequencing and annotation of LAB genomes are crucial not only for their functional application, but also for comparative genomics research. In this study, we selected two LAB strains, namely *L. casei* AP, a local strain isolated from the digestive tract of a healthy infant aged <1 month, and *L. plantarum* DR131, a LAB isolated from the indigenous fermented buffalo milk (*dadih*) of Indonesia, for genome sequencing based on Illumina technology and comparative analyses.

For *L. casei* AP, we assembled the genome sequences into 71 scaffolds (>500 bp) of a 2.95-Mb sequence, which represented approximately 96.72% of the entire genome, and annotated 2870 protein-coding genes at the genome level. For *L. plantarum* DR131, we assembled the sequence into 67 scaffolds (>500 bp) of a 4.44-Mb sequence, representing approximately 99.33% of the whole genome, and annotated 3069 protein-coding genes at the genome level. Junior et al. [37] reported the draft genome sequence of the *L. paracasei* strain DTA83 isolated from the stool sample of healthy infants in Rio de Janeiro (Brazil). The 2.8-Mb genome of this strain possessed 2825 protein-coding sequences distributed in 330 SEED subsystems. When we compared the results of our sequencing analysis, we found 98% similarity with the genome sequence of the *L. paracasei* strain DTA83 considering that they shared the same origin—the digestive tract of healthy infants. Patil et al. [38] studied the *L. plantarum* strain JDARSH, a potential probiotic with a wide range of functions isolated from sheep milk. The draft genome in our study was 3.20 Mb in size with 2980 protein-coding sequences. Comparison of the genome sequence of the *L. plantarum* DR131 used in our study with that of the *L. plantarum* strain JDARSH revealed a similarity of 97%. The predicted gene models of our sequenced genome and sequencing results were highly similar with JGI genome annotation, indicating that the quality of our sequence and annotation was reliable.

In the genome of *L. casei* AP, we only detected the genes *buk* (Scaffold14:33935:35056:+) and *ptb* (Scaffold14:33042:33938:+), which are involved in the butyric acid biosynthesis pathway for butanoate metabolism. These genes were not detected in *L. plantarum* DR131. In *L. casei* AP, the metabolic routes for butyrate production directly from glucose generate 2 mol H_2_/mol of butyrate in accordance with the equation C_6_H_12_O_6_ → butyric acid + 2H_2_ + 2CO_2_ [34]. Butyrogenesis proceeds through butyryl-coenzyme A (butyryl-CoA) generation from acetoacetyl-CoA via the intermediates β-hydroxybutyryl-CoA and crotonyl-CoA. Butyryl-CoA is then converted to butyrate via two pathways, one of which involves the generation of butyrate-phosphate via *ptb*, which is further converted to butyrate via *buk* [12].

Recently, medium-chain acyl–acyl carrier protein thio-esterase was proposed as the only terminal enzyme capable of producing butyric acid in *L. plantarum*. In addition to this enzyme, the enzymes present in *L. equisimilis* GGS 124 have been demonstrated to be capable of truncating the fatty acid biosynthesis pathway of FASII in engineered *E. coli* to release butyric acid and other medium-chain fatty acids [39]. Notably, the complementary activities of a medium-chain thio-esterase and FASII are also involved in the synthesis of hexanoic acid, octanoic acid, decanoic acid, dodecanoic acid, and tetradecanoic acid. Nevertheless, there is no explanation regarding the mechanism underlying butyric acid production in *L. plantarum.*

The enzymes and proteins involved in the fatty acid synthesis of FASII in *L. plantarum* DR131 (Table 2) can roughly be assigned to two categories: (i) those with highly conserved protein sequences and (ii) those with less conserved sequences, such as enoyl reductase, which comprises three discrete enzymes. Notably, except for *E. coli*, much of our discussions on proteins and genes were based on the DNA sequence data rather than on genetic or biochemical analyses [40]. The highly conserved proteins are often encoded within gene clusters.

Based on this study, we propose that *L. casei* AP employs the butanoate pathway as the main pathway in butyric acid synthesis. Meanwhile, butyric acid production in *L. plantarum* DR131 involves the GABA pathway in butanoate synthesis via an unknown mechanism, which was attributed to the complementary activities of medium-chain thio-esterase and FASII.

The above-mentioned analyses verify the outcomes of the previous experimental study regarding the capability of both LAB strains (*L. casei* AP and *L. plantarum* DR131) to synthesize butyric acid. The present study showed metabolic flexibility between species of the same lactobacilli genus in synthesizing the same organic acid. The data of genome sequencing revealed the metabolic pathways of butyric acid synthesis in two different species of lactobacilli, leading to a better understanding of how to optimize these pathways for butyric acid production.

## 5. Conclusions

*L. casei* AP and *L. plantarum* DR131 are capable of producing butyric acid. The metabolic pathways of butyrogenesis in these two strains are different. Both strains have potential for the development of metabolic engineering strategies.

## Figures and Tables

**Table 1:** KEGG annotation of the genes involved in butanoate metabolism in *Lactobacillus casei* AP.

KO	Abbreviation	Gene Names	Enzyme	Putative Gene
K01641	E2.3.3.10	hydroxymethylglutaryl-CoA synthase	2.3.3.10	LCAP_GM000043
K00626	E2.3.1.9, *atoB*	acetyl-CoA C-acetyltransferase	2.3.1.9	LCAP_GM000041
K00656	E2.3.1.54, pflD	formate C-acetyltransferase	2.3.1.54	LCAP_GM001343
K00929	*buk*	butyrate kinase	2.7.2.7	LCAP_GM001757
K04072	*adhE*	acetaldehyde dehydrogenase/alcohol dehydrogenase	1.2.1.10 1.1.1.1	LCAP_GM000697
K00135	*gabD*	succinate-semialdehyde dehydrogenase/glutarate-semialdehyde dehydrogenase	1.2.1.16 1.2.1.79 1.2.1.20	LCAP_GM002304
K00634	*ptb*	phosphate butyryltransferase	2.3.1.19	LCAP_GM001756
K01575	*alsD*, *budA*, *aldC*	acetolactate decarboxylase	4.1.1.5	LCAP_GM001865
K01652	E2.2.1.6L, *ilvB*, *ilvG*, *ilvI*	acetolactate synthase I/II/III large subunit	2.2.1.6	LCAP_GM001866
K00244	*frdA*	fumarate reductase flavoprotein subunit	1.3.5.4	LCAP_GM002528

**Table 2:** KEGG annotation of the genes involved in butanoate metabolism in *Lactobacillus plantarum* DR131.

KO	Abbreviation	Gene Names	Enzyme	Putative Gene
K01641	E2.3.3.10	hydroxymethylglutaryl-CoA synthase	2.3.3.10	LPDR_GM000252
K00656	E2.3.1.54, *pflD*	formate C-acetyltransferase	2.3.1.54	LPDR_GM001714
K00135	*gabD*	succinate-semialdehyde dehydrogenase/glutarate-semialdehyde dehydrogenase	1.2.1.16 1.2.1.79 1.2.1.20	LPDR_GM000712
K01575	*alsD*, *budA*, *aldC*	acetolactate decarboxylase	4.1.1.5	LPDR_GM000217
K01652	E2.2.1.6L, *ilvB*, *ilvG*, *ilvI*	acetolactate synthase I/II/III large subunit	2.2.1.6	LPDR_GM000843
K00244	*frdA*	fumarate reductase flavoprotein subunit	1.3.5.4	LPDR_GM001916
K04072	*adhE*	acetaldehyde dehydrogenase/alcohol dehydrogenase	1.2.1.10 1.1.1.1	LPDR_GM000307
K01580	E4.1.1.15, *gadB*, *gadA*, GAD	glutamate decarboxylase	4.1.1.15	LPDR_GM001627
K03778	*ldhA*	D-lactate dehydrogenase	1.1.1.28	LPDR_GM001493
K00016	LDH, *ldh*	L-lactate dehydrogenase	1.1.1.27	LPDR_GM002271
K00625	E2.3.1.8, *pta*	phosphate acetyltransferase	2.3.1.8	LPDR_GM001551
K00925	*ackA*	acetate kinase	2.7.2.1	LPDR_GM001960

**Table 3:** KEGG annotation of the genes involved in the complementary activities of a medium-chain thio-esterase and type 2 fatty acid synthase (FASII) in *Lactobacillus plantarum* DR131.

KO	Abbreviation	Gene Names	Enzymes	Putative Gene
K01071	MCH	medium-chain acyl-[acyl-carrier-protein] hydrolase	3.1.2.21	LPDR_GM001386
K00208	*fabI*	enoyl-[acyl-carrier protein] reductase I	1.3.1.9 1.3.1.10	LPDR_GM002661
K02372	*fabZ*	3-hydroxyacyl-[acyl-carrier-protein] dehydratase	4.2.1.59	LPDR_GM002650
K09458	*fabF*	3-oxoacyl-[acyl-carrier-protein] synthase II	2.3.1.179	LPDR_GM002655
K00059	*fabG*	3-oxoacyl-[acyl-carrier protein] reductase	1.1.1.100	LPDR_GM000931
K00645	*fabD*	[acyl-carrier-protein] S-malonyltransferase	2.3.1.39	LPDR_GM002653
K00648	*fabH*	3-oxoacyl-[acyl-carrier-protein] synthase III	2.3.1.180	LPDR_GM001441
K01962	*accA*	acetyl-CoA carboxylase carboxyl transferase subunit alpha	6.4.1.2	LPDR_GM002658
K02160	*accB*, *bccP*	acetyl-CoA carboxylase biotin carboxyl carrier protein	--	LPDR_GM001440
K01961	*accC*	acetyl-CoA carboxylase, biotin carboxylase subunit	6.4.1.2 6.3.4.14	LPDR_GM002658
K01963	*accD*	acetyl-CoA carboxylase carboxyl transferase subunit beta	6.4.1.2	LPDR_GM002658
K00997	*acpS*	holo-[acyl-carrier protein] synthase	2.7.8.7	LPDR_GM002257

## Data Availability

All data generated or analyzed during this study are included. The raw data are available from the corresponding author upon reasonable request.
